# Impact of nitrogen availability and soil communities on biomass accumulation of an invasive species

**DOI:** 10.1093/aobpla/plt045

**Published:** 2013-10-07

**Authors:** Devika Bajpai, Inderjit

**Affiliations:** Department of Environmental Studies, University of Delhi, Delhi 110007, India

**Keywords:** *Ageratina adenophora*, available nitrogen, exotic plant invasion, soil communities.

## Abstract

Exotic plant species impact belowground processes by influencing resource availability through enhanced microbial activity as a consequence of litter inputs. We have little understanding of the impact of microbe-driven nutrient fluctuations on the biomass accumulation of invasive species. Here we attempt to answer the question on whether soil community-driven nitrogen availability influences invader biomass. We discovered that soil communities cultured by *Ageratina adenophora*, a neotropical invader in Asia, retain available nitrogen that influences the biomass of the invader. Through soil manipulation experiments we found that *A. adenophora* grows better in soil with a higher available nitrogen content. *Ageratina adenophora*-invaded soil had higher microbial activity and available nitrogen due to higher inputs of terpene-rich litter compared with soil not yet invaded by it. Our results provide evidence that microbe-linked nitrogen availability exerts a positive impact on *A. adenophora* biomass accumulation. Our work emphasizes the importance of soil community-driven nitrogen availability in invasion success.

## Abstract (precis)

Exotic plant species impact belowground processes by influencing resource availability through enhanced microbial activity as a consequence of litter inputs. Soil communities cultured by *Ageratina adenophora*, a neotropical invader in Asia, retain available N that influence the growth of the invader. Invader triggers higher microbial activities through terpene-rich litter inputs to release nitrogen, which facilitates the invasion of *A. adenophora*. Our results provide evidence that microbial-linked nitrogen availability exert positive impact on *A. adenophora* biomass accumulation. Our work emphasizes the importance of soil communities-drive nitrogen availability in invasion success.

## Introduction

One of the questions that interest many ecologists is how nitrogen enables exotic plants to dominate ( Vitousek *et al.* 1987; Rout and Callaway 2009; Lee *et al.* 2012) and the role of belowground factors linked to it (Reinhart and Callaway 2004; Wardle *et al.* 2004; Gurevitch *et al.* 2011; Rout and Callaway 2012; Pendergast *et al.* 2013). Soil microbial communities are major components of the belowground ecosystem that influence exotic plant invasion by (i) making nutrients available through litter decomposition (Vitousek *et al.* 1987; Davis *et al.* 2000; Ehrenfeld 2003; Suding *et al.* 2013) and/or (ii) exerting negative or positive plant–soil feedback (Reinhart and Callaway 2006; Ehrenfeld 2010; Inderjit and Van der Putten 2010; Bever *et al.* 2012). These effects take place via impacts of either root exudates (Wardle *et al.* 2004; Ehrenfeld 2010; Lankau *et al.* 2011; Meisner *et al.* 2011; Perkins *et al.* 2011) or exotic plant litter on soil microbial activity (Ehrenfeld 2010; Meisner *et al.* 2012), resulting in the release or retention of nutrients that may promote invasion. In some cases, exotic litter accelerates nutrient cycling, particularly nitrogen (Cornwell *et al.* 2008). The decomposition of exotic litter largely depends on the amount of litter accumulated (Elgersma and Ehrenfeld 2011). It has been shown that litter of the invasive species *Berberis thunbergii* enhances net nitrogen mineralization and nitrification (Ehrenfeld *et al.* 2001; Elgersma and Ehrenfeld 2011). Exotic invasives impact microbial activities in soil, which stimulates microbial nitrogen cycling. For example, *Bromus tectorum* invasion in arid grasslands of southeastern Utah, USA, stimulates microbial nitrogen cycling by enhancing microbial activity (Schaeffer *et al.* 2012). Such fluctuations in resource availability have been reported to promote the invasibility of exotic invasive species (Davis and Pelsor 2001; Li and Stevens 2012; Mata *et al.* 2013).

We studied the role of nitrogen and its manipulation by soil microbial communities present in the invader's rhizosphere on the biomass accumulation of *Ageratina adenophora*, a neotropical invader in Asia (Inderjit *et al.* 2011; He *et al.* 2012). Biogeographic comparisons of native and introduced ranges of *A. adenophora* suggest that the invader exerts neutral to facilitative effects on plant species richness in its native range in Mexico, but had negative effects on plant species richness in its introduced ranges in China and India (Inderjit *et al.* 2011). The negative impact of *A. adenophora* on plant species richness in the introduced ranges was linked to biogeographic variations of terpenes released by *A. adenophora* litter, which suggested that it might be experiencing selection based on the composition of volatile organic compounds in its introduced ranges (Inderjit *et al.* 2011). *Ageratina adenophora* was reported to allocate greater amounts of nitrogen to photosynthesis (growth) and reduced allocation to cell walls (defence), thus resulting in an increase in its growth and vigour in the introduced ranges (Feng *et al.* 2009, 2011). This is also the case for *Microstegium vimineum* in the southern and eastern USA, which allocates more nitrogen to the aboveground parts than the belowground parts compared with native plants (Fraterrigo *et al.* 2011). A greenhouse study found that native soil communities had a more neutral than negative soil biota effect on *A. adenophora* relative to local species but soil biota from invaded sites had positive effects on the invader (Niu *et al.* 2007). However, it remains unclear whether nitrogen availability and microbial activity influence *A. adenophora* biomass accumulation. This is an important question in invasion ecology, where the effect of soil nutrients on invader performance is subject to debate (e.g. Funk and Vitousek 2007; Leishman *et al.* 2007, 2010; Heberling and Fridley 2013; Ordonez and Olff 2013), particularly under low nutrient availability conditions. Hence, the impact of *A. adenophora* on soil communities and microbial nitrogen retention needs further studies. *Ageratina adenophora* adds a large amount of leaf litter to the soil. We predict higher microbial activity in *A. adenophora* soils due to addition of terpene-rich litter, which may influence soil available nitrogen and thus the positive effects of *A. adenophora* biomass accumulation.

We hypothesized that higher availability of soil microbial-driven nitrogen exerts a positive impact on *A. adenophora* biomass accumulation. We designed a study to test our hypothesis by (i) comparing levels of available nitrogen and *A. adenophora* biomass accumulation in its non-sterilized and sterilized rhizosphere soil, (ii) manipulating the levels of nitrogen in soil to study the impact of nitrogen on *A. adenophora* biomass, and (iii) quantifying soil respiration and available nitrogen in soil collected from the *A. adenophora* rhizosphere and from areas not yet invaded by it (open areas).

## Methods

### Study area

The study was conducted in two sites situated in the foothills of the Himalayas, Palampur (32°7′0.12″N, 76°31′59.88″E; 1220 m a.s.l.) and Mussoorie (30°27′0″N, 78°4′48″E; 1825 m a.s.l.), which are invaded by *A. adenophora*. In each site we selected two microsites: a disturbed habitat and another undisturbed habitat. The first microsite in Palampur was a track of 25 km that occurred along a highway road (National Highway #21) outside the Institute of Himalayan Bioresources and Technology, and was considered a disturbed site (32°6′13.2″N, 76°33′20.6″E). Here, *A. adenophora* was present along roadside slopes with little natural sub-canopy vegetation along with a few trees of *Populus alba*, *Albezia lebbeck* and *Pinus roxburghii*. The second microsite was a closed forest 11 km from the Roadside site and was an undisturbed site (32°5′7.4″N; 76°31′4.8″E). Here *A. adenophora* was interspersed with trees like *P. alba*, *Gravillea robusta* and *Ficus virens*, and the ground was covered with *Cannabis sativa* and some grass species. In the Roadside site of Palampur, grass species like *Oplismenus* sp*.*, *Poa* sp*.* and *Cynodon* sp*.* were present. In addition to these species, plant species such as *Cirsium vulgare*, *Panicum clandestinum*, *Trifolium repens*, *Erigeron mucronatus* and *Oxalis corniculata* were found only in open areas. Other plant species like *Bidens pilosa* and *Lantana camara* were present in open areas as well as areas invaded by *A. adenophora*. In open areas of the forest site of Palampur, grass species such as *Oplismenus* sp*.* and *Cynodon* sp*.* and plants species like *Lathyrus aphaca*, *L. sphaericus*, *P. clandestinum*, *Lotus corniculatus*, *Briza minor* and *Anagallis arvensis* were found exclusively here, and species like *O. corniculata* and *Elettaria cardamomum* were present in both areas, open and invaded by *A. adenophora*.

In Mussoorie, the microsite with the disturbed habitat was a barren hill-slope (30°28′44.6″N; 78°3′9.1″E). Its original vegetation had been wiped out and it had nearly pure stands of *A. adenophora* with a few individuals of *Carex setigera*, *B. pilosa*, *E. mucronatus* and *Geranium mascatense.* The adjacent side of the Hill-slope site was an area not affected by hill slides and had a thick cover of vegetation dominated by species like *Quercus incana*, *Prunus cerasiodes* and *Crotoneaster bacillaris*. Owing to the complete destruction of the primary vegetation in the Hill-slope microsite in Mussoorie, no plant species was found exclusively in the open areas except for *Cynodon* sp. Species such as *Berberis aristata*, *Geranium lucidum*, *E. mucronatus*, *Crataegus crenulata*, *B. pilosa* and *Debregeasia hypoleuca* were, however, present in both open areas and areas invaded by *A. adenophora*. The second microsite in Mussoorie was a closed forest (30°28′12.6″N; 78°3′41.5″E) where *A. adenophora* was present inside a thick forest cover dominated by species such as *Q. incana*, *Rumex nepalensis*, *R. hastatus*, *E. mucronatus*, *Prinsepia utilis* and *B. aristata*. In the forest site of Mussoorie, plant species *Potentilla* sp., *Polytrichum* sp*.*, *Rosa macrophylla* and *Cirisium arvense* were present only in the open areas and *E. mucronatus*, *Rumex nepalensis*, *Smilax aspera*, *G. lucidum* and *T. repens* were found in both the open and *A. adenophora*-invaded areas.

### Soil effects

#### Impact of soil communities

To test the effect of soil communities of the *A. adenophora* rhizosphere, a total of 40 soil samples, 10 from each microsite, were collected from the rhizosphere of *A. adenophora* plants in Palampur and Mussoorie (10 plants × 2 microsites × 2 sites) in May 2012. Soil attached to *A. adenophora* roots was collected by pulling its roots and shaking off soil loosely bound to shallow roots, and identified as *A. adenophora* rhizosphere soil. In order to collect sufficient soil for one soil sample, ≈3–4 kg, 2–3 plants of *A. adenophora* were pulled. Soil was then air-dried and stored in paper bags for analysis.

In order to examine any effect of soil communities cultured by *A. adenophora* in its rhizosphere on its growth, we used non-sterile and sterile *A. adenophora* rhizosphere soil. Although soil sterilization is a widely employed technique to study the role of soil communities in plant invasion (e.g. Beckstead and Parker 2003; Callaway *et al.* 2004; Reinhart *et al.* 2005; Razavi darbar and Lakzian 2007; Rudgers and Orr 2009; Te Beest *et al.* 2009; Mangan *et al.* 2010; Kaur *et al.* 2012*a*; Birnbaum and Leishman 2013; Hendriks *et al.* 2013), the effect of nutrient leaching after sterilization cannot be ruled out. Half of each soil sample collected from the *A. adenophora* rhizosphere from the two sites was sterilized by autoclaving the soil at 121 °C and 103 kPa for 30 min three consecutive times. Each soil sample for sterilization was packed individually in a small bag and autoclaved followed by cooling at room temperature and then autoclaved again for three consecutive days, making a total of 80 soil samples (2 sites × 2 microsites × 10 plants × 2 treatments). Fifty grams of non-sterile or sterile *A. adenophora* rhizosphere soil were placed in each of 240 pots (2 sites × 2 microsites × 10 plants × 2 treatments × 3 experimental replicates) of 71.5 cm^3^ volume and irrigated with 15 mL of distilled water. *Ageratina adenophora* seeds collected from a single location in Mussoorie were sown in each pot. The seeds were allowed to germinate under controlled conditions of temperature (21/18 °C) and a day/night light regime of 12/12 h. Seedlings were thinned a week after full germination to five seedlings per pot. *Ageratina adenophora* plants including roots were harvested after 60 days of growth and dried at 50 °C for 3 days, and their biomass was measured as total dry weight of the whole plant and averaged per pot.

To study the impact of soil communities cultured by *A. adenophora* on available nitrogen, we analysed both non-sterile and sterile soil for available nitrogen. Fifty grams of dry soil were irrigated with 15 mL of distilled water and incubated at 22 °C for 5 days to make the soil conditions the same as when freshly collected from the field. Ten grams of soil were soaked in 20 mL of 0.5 M K_2_SO_4_, shaken for 1 h and filtered with nitrate-free Whatman #42. The concentration of available nitrogen was estimated as NO_3_-N using the colorimetric method (Anderson and Ingram 1993).

### Carbon (glucose) manipulation experiments

To examine the effect of nitrogen on *A. adenophora* biomass, we manipulated non-*A. adenophora* soil with different levels of carbon (C; glucose). An increase in microbial activity after the addition of labile C in the form of glucose results in nitrogen immobilization (Schmidt *et al.* 1997). One hundred and fifty grams of soil from the open area were taken in 160-cm^3^ pots and irrigated with 45 mL of 1.04, 2.08 or 4.17 mg glucose per mL, corresponding to 125, 250 and 500 µg C per g soil. Experimental conditions were temperature 21/18 °C and a 12/12 h day/night light regime. The soil was further amended with the same levels of glucose on the 30th day after the initiation of the experiment and terminated on the 60th day. Soil respiration was estimated on Day 0, and the 30th and 60th days, and available nitrogen was estimated on the 30th and 60th days from the start of the experiment from fresh soil samples. Soil respiration was measured as soil CO_2_ release by chemical titration (Anderson 1982). Fifty grams of fresh soil were taken in a 346.2-cm^3^ box, and a 5-cm-diameter Petri dish containing 10 mL of 0.1 N NaOH was placed on the soil inside the box and sealed to avoid any loss of CO_2_, and incubated for 24 h. One millilitre of 0.1 N BaCl_2_ was added to the NaOH to terminate incubation, and then titrated against 0.1 N HCl using phenolphthalein as an indicator. The amount of CO_2_ released was calculated following Anderson (1982). Available nitrogen in fresh soil was first extracted and analysed as described previously. Each soil sample was replicated five times for the analyses.

To examine the effect of available nitrogen, manipulated by adding glucose, on *A. adenophora* biomass, we sowed *A. adenophora* seeds and plants were allowed to grow for 60 days. Unamended soil irrigated with distilled water served as the control. There were a total of 20 pots (4 concentrations × 5 replicates). Soil was irrigated with a similar concentration of glucose after 30 days before thinning the seedlings to five seedlings per pot. The plants, both shoot and root, were harvested after 60 days. Each of the *A. adenophora* seedlings was weighed individually and their average/pot was obtained. They were dried at 50 °C for 3 days and their biomass estimated as their total dry weight.

### *Ageratina adenophora*-invaded soils versus open soils

Field soil was collected from the rhizosphere of *A. adenophora* or the area not yet invaded by it (identified as open soil) in two microsites in Palampur (Forest or Roadside) and Mussoorie (Forest and Hill-slope) in March 2011. *Ageratina adenophora* roots were pulled off and shaken to collect soil. Soil from the open areas in each microsite in two sites was collected at a depth of 10 cm. Sixteen soil samples from Palampur (2 areas (open and invaded) × 2 microsites × 4 subsites) and 20 soil samples from Mussoorie (2 areas × 2 microsites × 5 subsites) with each soil sample replicated three times were analysed for available nitrogen and soil respiration. Fifty grams of soil were first irrigated with 15 mL of distilled water and incubated at room temperature for 5 days to reactivate the microbial activity in the soil and then analysed for available nitrogen and soil respiration as described above.

### Data analysis

Differences in the mean dry weights of *A. adenophora* seedlings and available nitrogen in non-sterile and sterile soils were tested using the Independent Samples *t*-Test. To test the effect of site differences and sterilization treatment given to soil on the available nitrogen and the biomass of *A. adenophora* plants, we performed two-way ANOVA with treatment (non-sterile and sterile soil) and microsites (disturbed and forest sites) as fixed factors, for both Palampur and Mussoorie.

To see the effect of manipulation of microbial populations of soil communities when the soils were treated with different levels of C on *A. adenophora*, we tested the correlation between the concentrations of C added to the soil in the form of glucose and available nitrogen, and between C concentrations and soil respiration. Pearson's correlation coefficient *r* was calculated to find out the correlation between soil C and available nitrogen or soil respiration. The effect of addition of different levels of C on *A. adenophora* plant biomass was tested by calculating the differences in the biomass of *A. adenophora* plants grown in C-treated soil at each concentration and between those grown in untreated soil taken as the control using the Independent Samples *t*-Test.

Differences in the soil respiration or available nitrogen in the open area and *A. adenophora* rhizosphere soil were tested using the Independent Samples *t*-Test (*P* < 0.05) except for the differences in soil respiration in Mussoorie forest where the data were not normally distributed (Shapiro–Wilk test, *P* > 0.05) and were tested using the Mann–Whitney *U* test (asymptotic significance two-tailed, *P* < 0.05). All analyses were carried out using SPSS 16.0 (SPSS Inc. 2008).

## Results

### Soil effects

**Figure 1.**
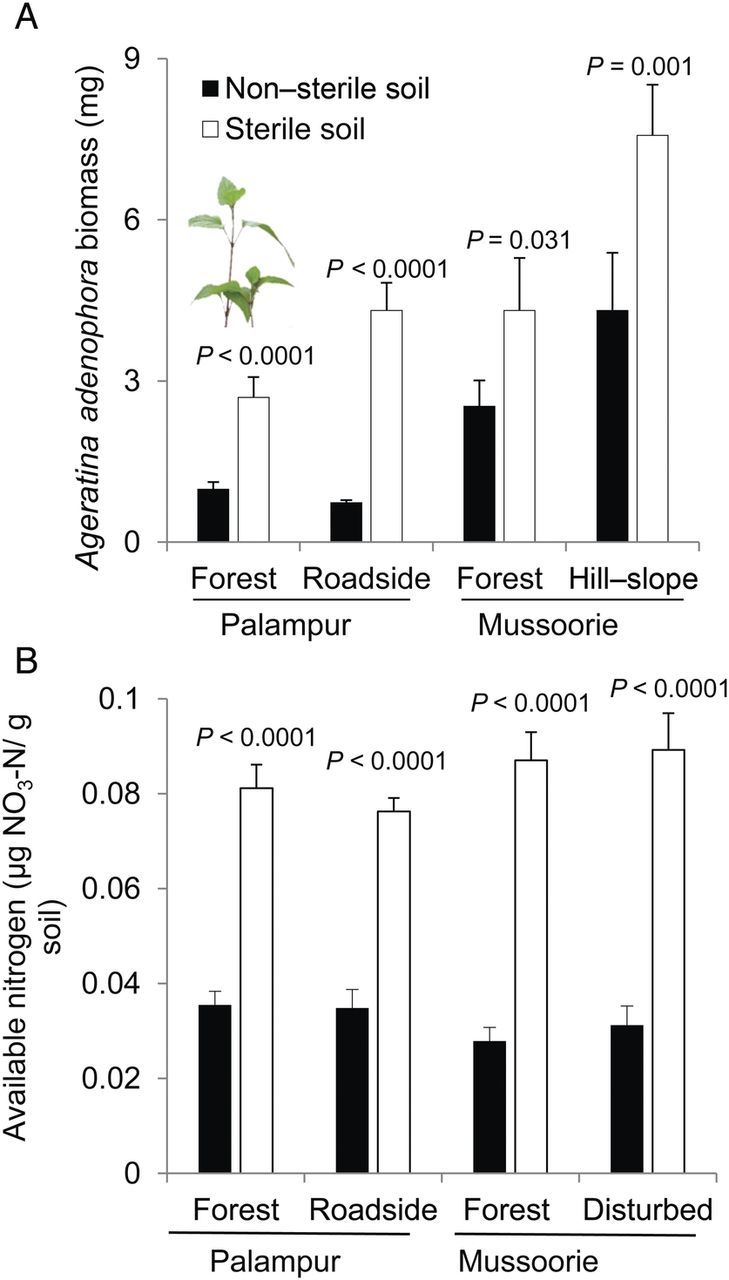
(A) Biomass accumulation (mg) of 60-day-old *A. adenophora* plants grown in the non-sterile and sterile rhizosphere soil of *A. adenophora* from Palampur and Mussoorie. Soil from the rhizosphere of *A. adenophora* was collected from undisturbed (Forest) and disturbed (Roadside or Hill-slope) habitats. (B) Available nitrogen (µg NO_3_-N per g soil) in soil from the rhizosphere of *A. adenophora* in Palampur and Mussoorie or in the sterilized treatment of the same. Error bars represent **+1 SE** of the mean. *P* values above the bars represent a significant difference between *A. adenophora* biomass accumulation (A) or available nitrogen (B) in the two different soil treatments (Independent Samples *t*-Test; *P* < 0.05).

We observed higher *A. adenophora* biomass accumulation when it was grown in the sterile treatment of *A. adenophora* rhizosphere soil compared with non-sterile rhizosphere soil in both sites (Fig. 1A). In Palampur, the difference in *A. adenophora* biomass in sterile soil was 174.7 % (df = 58, *t* = −6.902, *P* < 0.0001) in the Forest site and 487.89 % (df = 58, *t* = −9.670, *P* < 0.0001) in the Roadside site (Fig. 1A). In Mussoorie, we observed a difference of 70.8 % in the Forest site (df = 58, *t* = −2.216, *P* = 0.031) and 75.7 % in the Hill-slope site (df = 58, *t* = −3.352, *P* = 0.001) in *A. adenophora* biomass when it was grown in sterile soil compared with non-sterile soil (Fig. 1A). The differences in available nitrogen in sterile soil compared with non-sterile soil were 129.4 % (df = 18, *t* = −7.953, *P* < 0.0001) in the Forest site, Palampur, and 119.6 % (df = 18, *t* = −8.426, *P* < 0.0001) in the Roadside site. In Mussoorie, the differences in available nitrogen were 213.66 % (df = 18, *t* = −8.958, *P* < 0.0001) in the Forest site and 186.9 % (df = 18, *t* = −6.600, *P* < 0.0001) in the Hill-slope site (Fig. 1B).

Two-way ANOVA was carried out with soil (non-sterile and sterile) and microsites (disturbed and forest) as fixed factors, to see whether there is any effect of variation in the sites on the differences in the biomass of *A. adenophora* plants and available nitrogen in soil after sterilization treatment (Table 1). The effect of the two factors as well as their interactions was significant when *A. adenophora* was grown in soil collected from its rhizosphere from Palampur. In Mussoorie, the effect of the two fixed factors was also significant but the effect of their interactions was not (Table 1). In the case of available nitrogen present in the rhizosphere soil of *A. adenophora*, in Palampur as well as in Mussoorie only the effect of the factor Soil was significant (Table 1).

**Table 1.** Summary of two-way ANOVA showing the effects of soil (non-sterile and sterile) and microsite (disturbed and forest) and their interaction on *A. adenophora* biomass accumulation, and on the available nitrogen in the rhizosphere soil from Palampur and Mussoorie. d.f., degrees of freedom. Significance level (in bold): *P < 0.05.

Source of variation	Biomass of *A. adenophora*			Available nitrogen		
	d.f.	*F*	*P* value	d.f.	*F*	*P* value
Palampur						
Soil	1	141.029	<0.0001*	1	132.738	<0.0001*
Microsite	1	9.384	0.003*	1	0.536	0.469
Soil × microsite	1	17.457	<0.0001*	1	0.312	0.580
Mussoorie						
Soil	1	15.913	<0.0001*	1	113.590	<0.0001*
Microsite	1	15.954	<0.0001*	1	0.254	0.618
Soil × microsite	1	1.359	0.246	1	0.011	0.917

### Carbon (glucose) manipulation studies

**Figure 2.**
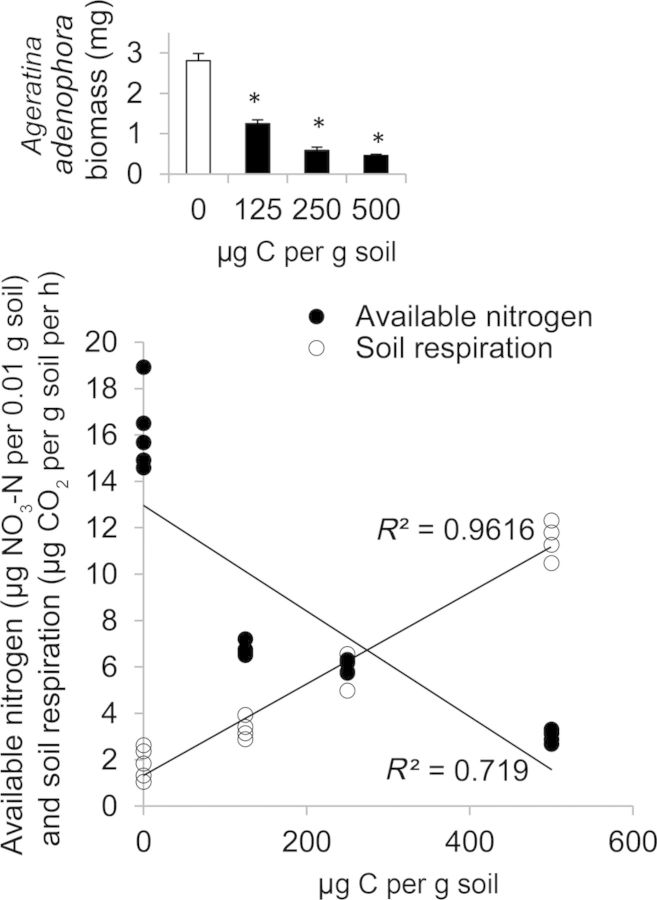
Correlation between C concentration (µg C per g soil) and available nitrogen (µg NO_3_-N per 0.01 g soil, solid spheres) or soil respiration (µg CO_2_ per g soil per h, hollow spheres), in soil from open areas treated with four levels of C, control (0 µg C per g soil), low (125 µg C per g soil), medium (250 µg C per g soil) and high (500 µg C per g soil) on the 60th day of first amendment. Double asterisks indicate that correlation coefficient *r* is significant at *P* < 0.01. Inset, mean (**+SE**) biomass of 60-day-old *A. adenophora* seedlings in soil from open areas treated with the above-mentioned three levels of C on the 60th day of first amendment. Asterisks above the bars represent a significant difference between the dry biomass of seedlings grown in soils amended with glucose and the unamended soil taken as the control (Independent Samples *t*-Test; *P* < 0.05, Table 2).

We treated soil from the open area with different levels of C (glucose) to study its effect on *A. adenophora* biomass, soil respiration and available nitrogen. We observed an increase in available nitrogen levels in the control (0 μg C per g soil) soil after 30 (9.84 ± 0.73 μg C per g soil) or 60 (16.11 ± 0.77 μg C per g soil) days compared with 0 (1.84 ± 0.17 μg C per g soil) days from the start of the experiment. Soil respiration in the control soil, however, was decreased on 30 (2.08 ± 0.13 μg CO_2_ per g soil per h) or 60 (1.83 ± 0.29 μg CO_2_ per g soil per h) days compared with 0 (4.76 ± 0.15 μg CO_2_ per g soil per h) days from the start of the experiment. A significant positive correlation was observed between the C concentrations in soil and soil respiration (*N* = 20, *r* = 0.981**, *P* < 0.0001, Fig. 2) and a significant negative correlation between the C concentrations and available nitrogen in the soil (*N* = 20, *r* = −0.848**, *P* < 0.0001, Fig. 2). We observed that *A. adenophora* biomass was significantly lower in soil modified with different levels of C (glucose) compared with the untreated control (Fig. 2, inset; Table 2).

**Table 2.** Summary of the Independent Samples *t*-Test conducted for differences in *A. adenophora* biomass accumulation when grown in soil treated with 0 µg C per g soil (identified as the control) or glucose (125, 250 or 500 µg C per g soil). d.f., degrees of freedom. Significance level (in bold): *P < 0.05.

Between C concentrations (µg C per g soil)	d.f.	*F*	*T*	*P* value
0 and 125	8	3.603	7.527	<0.0001*
0 and 250	8	4.684	10.977	<0.0001*
0 and 500	8	13.218	12.579	<0.0001*

### *Ageratina adenophora*-invaded soils versus open soils

**Figure 3.**
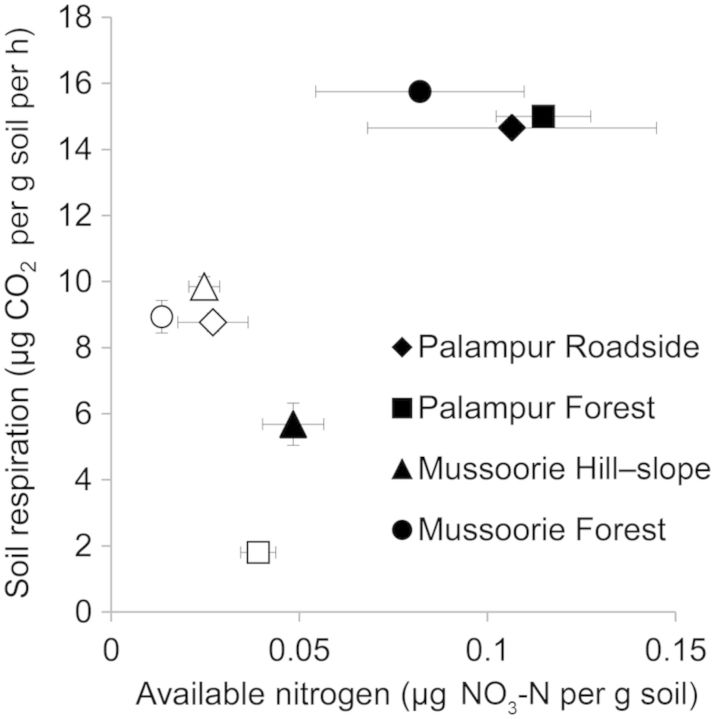
Available nitrogen and soil respiration in soils from open areas (hollow) and the rhizosphere of *A. adenophora* (solid) in microsites: Forest (square, *n* = 4) or Roadside (diamond, *n* = 4) in Palampur, and Forest (circle, *n* = 5) or Hill-slope (triangle, *n* = 5) in Mussoorie: Bars indicate ±1 SE. Differences between available nitrogen and soil respiration in soil from open areas and the rhizosphere of *A. adenophora* from a site were tested using the Independent Samples *t*-Test (*P* < 0.05) except for the differences in soil respiration in Mussoorie forest where the data were not normally distributed (Shapiro–Wilk test, *P* > 0.05) and were tested using the Mann–Whitney *U* test (asymptotic significance two-tailed, *P* < 0.05).

Higher values of soil respiration and available nitrogen were observed in *A. adenophora*-invaded soils compared with the open areas (Fig. 3). The exception was the Hill-slope site in Mussoorie where a higher value of soil respiration in soils from open areas was observed compared with soil collected from the rhizosphere of *A. adenophora* (Fig. 3). Higher values of soil respiration in the *A. adenophora* rhizosphere in Palampur compared with open soil were observed: by 67.1 % (Roadside, df = 22, *t* = −19.651, *P* < 0.0001) and 732.45 % (Forest, df = 22, *t* = −65.81, *P* < 0.0001) (Fig. 3). In Mussoorie, soil respiration in the *A. adenophora* rhizosphere was 76.23 % higher compared with open soil (Forest, *N* = 15, *P* < 0.0001, Shapiro–Wilk test). However, we observed lower values of soil respiration in *A. adenophora* rhizosphere soils from the Hill-slope site in Mussoorie: by 42.31 % (df = 28, *t* = 5.931, *P* < 0.0001) compared with open areas (Fig. 3).

We observed that the available nitrogen in the soil collected from the *A. adenophora* rhizosphere was also significantly higher compared with that in soil collected from open areas in Palampur as well as Mussoorie. It was 293.9 % (Roadside, df = 22, *t* = −3.801, *P* = 0.001) and 193.96 % (Forest, df = 22, *t* = −9.162, *P* < 0.0001) higher in the *A. adenophora* rhizosphere soils in Palampur. In Mussoorie, we observed an increase in the levels of available nitrogen in the *A. adenophora* rhizosphere of 95.62 % in the Hill-slope site (df = 28, *t* = −4.450, *P* < 0.0001) and 510.17 % in the Forest site (df = 28, *t* = −4.467, *P* = 0.001) compared with open soils (Fig. 3).

## Discussion

We found that sterilization of *A. adenophora* rhizosphere soil exerts a positive impact on its biomass accumulation and resulted in higher levels of available nitrogen (Fig. 1). The higher biomass accumulation of *A. adenophora* is attributed to a post-sterilization nitrogen release (Fig. 1B). This could also be due to lower microbial activity (Fig. 2) or could also be related to a release from soil pathogens. Soil microbes retain inorganic nitrogen and release it back to the soil upon sterilization (Razavi darbar and Lakzian 2007). The higher biomass accumulation of *A. adenophora* and higher levels of available nitrogen in sterile soil than in non-sterile *A. adenophora* soil (Fig. 1) suggest that higher nitrogen availability has positive effects on *A. adenophora* biomass accumulation. By manipulating microbial activity, we were able to determine the effect of nitrogen availability on *A. adenophora* biomass accumulation. We manipulated the levels of nitrogen by amending soil with four levels of glucose. Addition of glucose as a C source in soil lowers the nitrogen availability by enhancing microbial activity (Brady and Weil 2008). Soil sterilization experiments showed a higher biomass increase in sterile soils (Fig. 1), which could be explained by addressing the effect of microbial activity (due to a lower activity of microbes on sterile and non-enriched soils) on biomass accumulation and nitrogen availability (Fig. 2). A significant negative correlation between the glucose-driven CO_2_ release by soil communities and available nitrogen suggests that higher microbial activity lowers the levels of nitrogen (Fig. 2). We also observed that *A. adenophora* biomass was significantly lower in soil modified with different levels of C (glucose) compared with the untreated control (Fig. 2, inset; Table 2). The observed glucose dose-dependent suppression of *A. adenophora* biomass in glucose-amended soil can be linked to dose-dependent lowering of nitrogen (Fig. 2, inset). This study suggests that *A. adenophora* grows better in soils with higher nitrogen levels. Since *A. adenophora* allocates higher amounts of nitrogen to its growth in introduced ranges than in native ranges where it allocates more nitrogen to defence (Feng *et al.* 2009, 2011), and forms mono-dominant communities in introduced ranges (Inderjit *et al.* 2011), the higher levels of nitrogen in *A. adenophora* soils compared with open areas contribute to *A. adenophora* biomass accumulation (see also Fig. 2, inset).This adds to the growing evidence in the literature that available nitrogen facilitates the growth of exotic invasives.

Our data on higher levels of nitrogen in *A. adenophora* soils compared with open soils could be explained by the higher build-up of organic C (3.3 %) below its canopies compared with open forest soil (0.8 %), which could be due to higher *A. adenophora* litterfall (non-forest area, 1.4–10.6 g m^−2^; forest area, 4.5–17.6 g m^−2^). *Ageratina adenophora* litter releases a variety of mono- and sesquiterpenes into the environment (Inderjit *et al.* 2011). Terpenes get adsorbed onto soil particles (Muller and del Moral 1966). Accumulation of terpene-rich *A. adenophora* litter results in higher microbial activity, leading to the release of nitrogen from *A. adenophora* litter. Higher accumulation of organic C triggers enhanced microbial activity that results in nitrogen release from litter (Brady and Weil 2008). The litter of certain exotic species is known to release higher amounts of nitrogen compared with native litter in spite of similar decomposition rates (Hata *et al.* 2012; Meisner *et al.* 2012). For example, certain exotic species accumulate greater amounts of nutrients as a consequence of greater organic C below their canopies and in their rhizosphere, e.g. *Artemisia frigida* and *Caraganda microphylla* (Su *et al.* 2004; Ehrenfeld *et al.* 2005) or trees like *Prosopis juliflora*, an aggressive invasive (Kaur *et al.* 2012*b*). Higher levels of nitrogen-fixing bacterial communities are reported in the *A. adenophora* rhizosphere compared with open soils (Xu *et al.* 2012) but nitrogen levels in the present study seem to link with microbial activities as evident from higher nitrogen levels in sterilized *A. adenophora* rhizosphere soil (Fig. 1B). We compared *A. adenophora* and non-*A. adenophora* (open) soils in order to understand its impact on belowground processes largely because such comparisons would include long-term changes in nutrient and litter dynamics (Meisner *et al.* 2011). Our work suggests that the invader could trigger higher microbial activities through litter inputs, which facilitate nitrogen release. Any role of soil communities needs to be integrated with litter quality and resource availability. There is a need to focus our future work on studying whether local species co-occurring with the invader also share the extra benefit that exotic species gain from the available nitrogen. This would help us to understand how nitrogen enables exotic plants to dominate.

## Conclusions

Our results show that *A. adenophora* biomass increase is affected by nitrogen availability and that this is due to its rhizosphere retaining nitrogen. The importance of nitrogen in facilitating *A. adenophora* biomass is established by comparing its growth in non-sterile and sterile soil from its rhizosphere. We therefore consider it important to study the impact on nitrogen availability when examining the role of soil communities in plant invasion (Perkins and Nowak 2013). Our results are also important for conservation biologists because we show that nitrogen facilitates invasion of exotic species like *A. adenophora* through soil community. Any fertilization in the forest should be reviewed, particularly for the areas invaded by exotic species with potential soil community-driven nitrogen availability as a mechanism of success (Perry *et al.* 2010).

## Sources of Funding

The research was funded by the Council of Scientific & Industrial Research (CSIR) and the University of Delhi.

## Contributions by the Authors

D.B. and Inderjit designed the research. D.B. performed the experiments. D.B. and Inderjit analysed the data and wrote the paper.

## Conflicts of Interest Statement

None declared.
