# Corrigendum

**DOI:** 10.1093/aobpla/plu005

**Published:** 2014-02-20

Bajpai D, Inderjit. 2013. Impact of nitrogen availability and soil communities on biomass accumulation of an invasive species. *AoB PLANTS* **5**: plt045; doi:10.1093/aobpla/plt045

The author has supplied the following corrected version of Figure 2.

**Figure 2.**
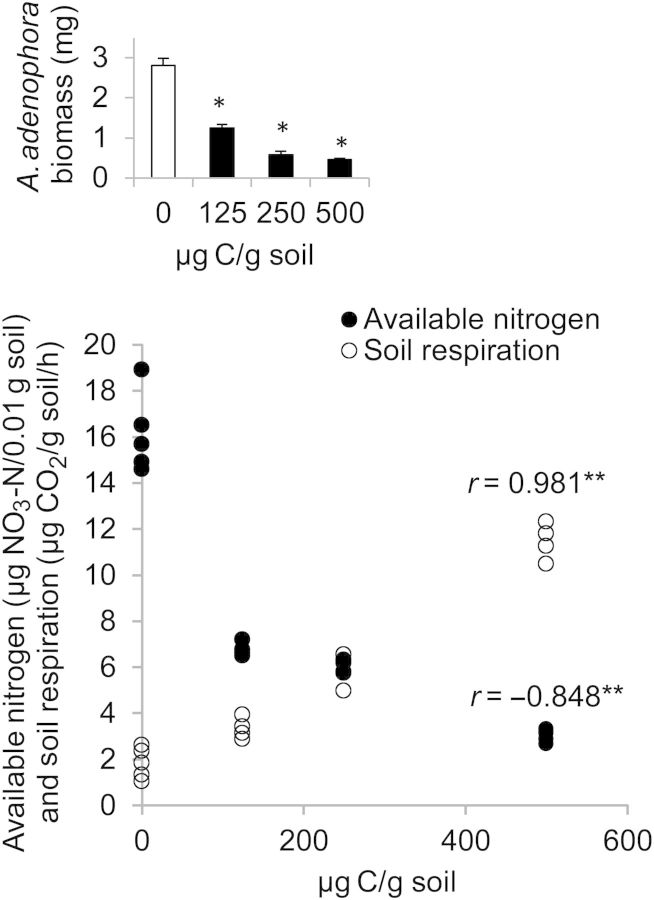
Correlation between C concentration (µg C per g soil) and available nitrogen (µg NO_3_-N per 0.01 g soil, solid spheres) or soil respiration (µg CO_2_ per g soil per h, hollow spheres), in soil from open areas treated with four levels of C, control (0 µg C per g soil), low (125 µg C per g soil), medium (250 µg C per g soil) and high (500 µg C per g soil) on the 60th day of first amendment. Double asterisks indicate that correlation coefficient *r* is significant at *P* < 0.01. Inset, mean (+SE) biomass of 60-day-old *A. adenophora* seedlings in soil from open areas treated with the above-mentioned three levels of C on the 60th day of first amendment. Asterisks above the bars represent a significant difference between the dry biomass of seedlings grown in soils amended with glucose and the unamended soil taken as the control (Independent Samples *t*-Test; *P* < 0.05, Table 2).

